# Joint RNA-Seq and miRNA Profiling Analyses to Reveal Molecular Mechanisms in Regulating Thickness of Pod Canopy in *Brassica napus*

**DOI:** 10.3390/genes10080591

**Published:** 2019-08-05

**Authors:** Zhiyou Chen, Qiang Huo, Hong Yang, Hongju Jian, Cunmin Qu, Kun Lu, Jiana Li

**Affiliations:** 1 College of Agronomy and Biotechnology, Southwest University, Chongqing 400715, China; 2 Academy of Agricultural Sciences, Southwest University, Chongqing 400715, China; 3 Engineering Research Center of South Upland Agriculture, Ministry of Education, Chongqing 400715, China; 4 State Cultivation Base of Crop Stress Biology for Southern Mountainous Land of Southwest University, Chongqing 400715, China; 5 College of Life Sciences, Yangtze University, Jingzhou 434025, China

**Keywords:** microRNA (miRNA), thickness of pod canopy, *Brassica napus*, nitrogen-related pathway

## Abstract

Oilseed rape (*Brassica napus*) is the second largest oilseed crop worldwide. As an architecture component of *B. napus*, thickness of pod canopy (TPC) plays an important role in yield formation, especially under high-density cultivation conditions. However, the mechanisms underlying the regulation of TPC remain unclear. RNA and microRNA (miRNA) profiling of two groups of *B. napus* lines with significantly different TPC at the bolting with a tiny bud stage revealed differential expressions of numerous genes involved in nitrogen-related pathways. Expression of several nitrogen-related response genes, including *ASP5*, *ASP2*, *ASN3*, *ATCYSC1*, *PAL2*, *APT2*, *CRTISO*, and *COX15*, was dramatically changed in the thick TPC lines compared to those in the thin TPC lines. Differentially expressed miRNAs also included many involved in nitrogen-related pathways. Expression of most target genes was negatively associated with corresponding miRNAs, such as miR159, miR6029, and miR827. In addition, 12 (including miR319, miR845, and miR158) differentially expressed miRNAs between two plant tissues sampled (stem apex and flower bud) were identified, implying that they might have roles in determining overall plant architecture. These results suggest that nitrogen signaling may play a pivotal role in regulating TPC in *B. napus*.

## 1. Introduction

**Figure 1:**
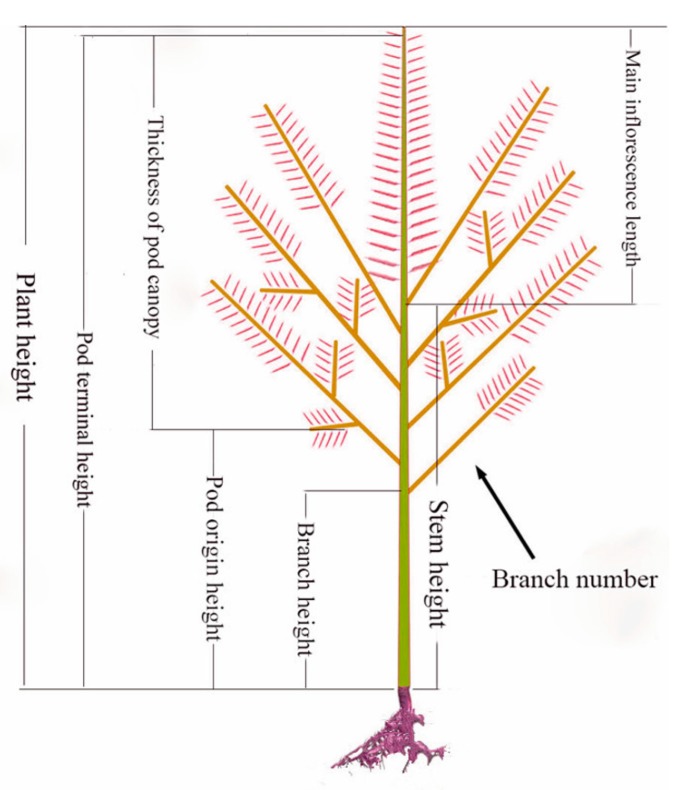
The thickness of pod canopy (TPC) related to architecture traits in *Brassica napus*.

Oilseed rape (*Brassica napus*, 2*n* = 38, AACC) is one of the most important oil crops in the world. With the ongoing decreases in arable land and increases in human population, crop yield improvement is a highly important goal. In domesticated crops, one strategy to increase yield is to focus on ideal-type breeding, i.e., plant architecture optimization, which has proved to be useful for improving crop adaptability to different environments and increasing seed and fruit yield [1,2,3,4,5,6]. Thickness of pod canopy (TPC), defined as the thickness of the plant canopy from the bottom-most effective (seed-bearing) pod to the uppermost effective pod, is a key trait determining the three-dimensional structure of the *B. napus* plants, with direct relationships with other important traits (Figure 1).

Plant architecture (PA) optimization is fundamental for crop yield improvement, but the genetic basis of many architectural traits in *B. napus* remains unknown. PA modifications play significant roles in crop adaptation and yield potential [7,8]. Over the past two decades, numerous genes involved in plant phenotypic characteristics have been investigated to unravel their molecular regulatory mechanism. Many of these are related to phytohormone biosynthesis and signaling pathways. For example, genes regulating plant height are involved in gibberellin (GA) metabolism and signal transduction [5,9]. Genes involved in auxin (including indoleacetic acid (IAA)) biosynthesis and signaling pathways have been found to function as a master switch in the shoot apical meristem (SAM) initiation and development, which is required to initiate new branches, leaves, and flowers [10,11,12]. Moreover, several genes related to the metabolic regulation and transport of three phytohormones, brassinosteroid (BR), cytokinin (CK), and strigolactone (SL), have also been proved to be important in controlling PA-related traits, such as *D27*, *D14*, *BRI1*, *BKI1*, *KN1*, and C*KX* [13,14,15,16,17,18]. Although several genes involved in PA regulation and development have been characterized in diverse plant species, understanding of morphogenesis and developmental regulation in *B. napus* is still limited.

MicroRNAs (miRNAs) are 20–24 nt small non-coding endogenous RNAs that repress the expression of a wide range of target genes in animals and plants [19]. In plants, mature miRNAs are generated via a multistep enzymatic process. Briefly, the primary miRNA transcripts (pri-miRNAs) are firstly synthesized by RNA polymerase II (Pol II) and further form imperfect foldback hairpin structures, which are then processed into a stem-loop precursor (pre-miRNAs), additionally excised to form miRNA/miRNA* duplexes. Eventually, mature miRNAs are released from these duplexes and then combined with RNA-induced silencing complexes (RISCs) to regulate their target genes either by repressing translation or miRNA cleavage [20,21].

Increasing evidence shows that miRNAs are involved in diverse phenotypes, such as plant growth, development processes, and stress responses. Nine conserved miRNA families, miR156, miR159, miR160, miR164, miR166/165, miR167, miR169, miR172, and miR319, play key roles in flower development [22]. MiR824, miR319, miR390, and miR396 are critical in regulating leaf development [23,24,25]. MiR166 regulates plant vascular development through decreasing the expression level of the target gene, a Homeodomain-leucine zipper (HD-ZIP) transcription factor *ATHB15* [26]. MiR160 targets auxin-response factors (ARFs) to control fertility and root cap formation in Arabidopsis [27]. MiR159 acts as a major connection among at least three phytohormones, GA, abscisic acid (ABA), and ethylene (ET), and regulates developmental processes involving anther development by targeting the GA-regulated myeloblastosis (GAMYB) genes [28,29]. MiR172 and miR156 conduct the vegetative phase change [30]. MiR172 controls the flowering time and floral organ identity by targeting APETELA2 (AP2) transcription factors [31,32,33].

In recent years, many studies have shown the interaction between miRNAs and plant architecture formation. Overexpression of miR319 influences internode elongation and results in a reduction of plant height of rice (*Oryza sativa*) [34]. MiR160 targeting ARF and miR166 targeting ABA-responsive-element binding factors, elevated the expression of *GH3* and ABA-related genes and decreased the transcript level of *SAUR*, thereby reducing the plant height in cotton (*Gossypium hirsutum*) [35]. MiR171c negatively regulates shoots branching through the cleavage of *GRAS* genes, *SCARECROW-LIKE6-II* (*SCL6-II*), *SCL6-III*, and *SCL6-IV* [36]. MiR156 and miR172 regulate the rice tiller and panicle branching by targeting *SQUAMOSA PROMOTER BINDING PROTEIN-LIKE* (*SPL*) and *AP2* transcription factors, respectively [37]. More recently, several miRNAs, including miR156, miR172, and miR319, have also been found to regulate branch angles by targeting genes involved in auxin and BR signaling in *B. napus* [38].

In this study, we performed RNA sequencing (RNA-seq) and miRNA expression profiling using high-throughput sequencing for two sets of lines with significantly divergent TPC in order to gain an insight into the mechanism controlling TPC variation in *B. napus*, and we subsequently analyzed the relationships between the expression of miRNAs and their targets. Our results provide insights into the mechanisms underlying the TPC regulation in *B. napus*, which should aid the improvement of this plant crop through breeding for optimized plant architecture.

## 2. Materials and Methods

### 2.1. Plant Materials, RNA Sequencing, and Small RNA Sequencing

Plant samples used for the miRNA and RNA-seq analyses were cultivated at the Chongqing Rapeseed Engineering Research Center, Southwest University, Chongqing, China (106.40° E, 29.80° N). Three years of continuous observation showed that *B. napus* lines, YC4 (SWU71) and YC33 (10–1047), had relatively higher TPC, while YC11 (Zhongshuang11) and YC15 (Zhongyou821) possessed relatively lower TPC (Table S1). The four lines were therefore selected for comparative analyses. Two tissues, the stem apex and nearby tiny flower buds, were collected from four selected lines at the bolting with the tiny flower bud stage, which is a vital phase for the TPC formation (after this stage, the stems elongate and pollens begin to develop) in *B. napus*. For each sample, two biological replicates, each harvested from five independent plants, were collected for sequencing and quantitative real-time PCR (qRT-PCR) analysis. Total RNA was isolated using Trizol Reagent (Invitrogen, USA), according to the manufacturer’s protocols. After quality evaluation of the RNA, using gel electrophoresis [39], RNA samples were sent to the Novogene Corporation (Beijing, China) for construction of RNA and small RNA (sRNA) sequencing libraries.

A total amount of 1.5 μg RNA per sample was used as the input for the RNA sample preparations. Sequencing libraries were generated using the NEBNext Ultra RNA Library Prep Kit for Illumina (NEB, San Diego, CA, USA). Briefly, miRNA was purified from total RNA using poly-T oligo-attached magnetic beads. Fragmentation was carried out using divalent cations under an elevated temperature in the NEBNext First Strand Synthesis Reaction Buffer (5X). First strand cDNA was synthesized using a random hexamer primer and M-MuLV Reverse Transcriptase. Second strand cDNA synthesis was subsequently performed using DNA Polymerase I and RNase H. Remaining overhangs were converted into blunt ends via exonuclease/polymerase activities. After adenylation of 3′ ends of DNA fragments, the NEBNext Adaptor with a hairpin loop structure was ligated to prepare for hybridization. To select cDNA fragments with the right length, the library fragments were purified with an AMPure XP system (Beckman Coulter, Beverly, WV, USA). A 3 μL USER Enzyme (NEB, USA) was then used with size-selected, adaptor-ligated cDNA at 37 °C for 15 min, followed by 5 min at 95 °C before PCR. PCR was then performed with Phusion High-Fidelity DNA polymerase, Universal PCR primers, and an Index (X) Primer. Finally, products were purified, and the library quality was assessed using the Agilent Bioanalyzer 2100 (Agilent Technologies, Palo Alto, CA, USA).

A total amount of 3 μg total RNA per sample was used as input material for the small RNA library. Sequencing libraries were generated using the NEBNext Multiplex Small RNA Library Prep Set for Illumina (NEB, USA). Briefly, the NEB 3′ SR Adaptor was directly and specifically ligated to the 3′ end of the miRNA, small interfering RNAs (siRNA), and Piwi-interacting RNA (piRNA). After the 3′ ligation reaction, the SR RT Primer hybridized to the excess of the 3′ SR Adaptor (that remained free after the 3′ ligation reaction) and transformed the single-stranded DNA adaptor into a double-stranded DNA molecule. Double-strand DNAs (dsDNAs) are not substrates for a ligation mediated by T4 RNA Ligase 1 and therefore do not ligate to the 5′ SR Adaptor in the subsequent ligation step. A 5′-ends adapter was ligated to the 5′-ends of miRNAs, siRNA, and piRNA. The first strand cDNA was then synthesized using M-MuLV Reverse Transcriptase. PCR amplification was performed using a LongAmp *Taq* 2X Master Mix, a SR Primer for Illumina, and an index (X) primer. PCR products were purified on a polyacrylamide gel. DNA fragments corresponding to 140~160 bp (the length of small noncoding RNA plus the 3′ and 5′ adaptors) were recovered and dissolved in an 8 μL elution buffer. At last, library quality was assessed on the Agilent Bioanalyzer 2100 (Agilent Technologies, Palo Alto, CA, USA).

### 2.2. Transcriptome Analysis

Raw sequencing data was firstly filtered by removing adaptors and low-quality reads and the clean data was then mapped onto the *B. napus* reference genome v4.1 (http://www.genoscope.cns.fr/brassicanapus/). Cufflinks (http://cole-trapnell-lab.github.io/cufflinks/cuffdiff/index.html) were employed to calculate expression levels of genes and miRNAs based on fragments per kilobase of exon per million fragments mapped (FPKM) values [40]. Novel genes were identified from transcripts newly assembled from Cufflinks results, and those encoding peptides with less than 50 amino acid residues were removed [41]. DEseq 1.36.0 (http://www.bioconductor.org/packages/release/bioc/html/DESeq.html) was used to identify genes differentially expressed between high- and low-TPC lines in *B. napus* [42]. Differences in gene expression levels between the two group lines were determined from the ratio of corresponding FPKM values. The false discovery rate (FDR) was estimated to correct the *P*-values and detect the statistical significance of differences. Thresholds FDR ≤ 0.01 and the absolute value of the log_2_ ratio ≥ 2 were used for identification of differentially expressed genes (DEGs). Functional annotation was performed by using BLASTX to compare proteins against three protein databases: The Kyoto Encyclopedia of Genes and Genomes (KEGG), the Swiss-Prot, and the National Center for Biotechnology Information NCBI protein database. The Blast2GO program was used for gene ontology (GO) annotation, and all annotated genes were aligned to the database and then the gene numbers associated with each GO term were obtained [43]. The annotations were then refined and enriched using the R package TopGO 2.36.0 (http://www.bioconductor.org/packages/release/bioc/html/topGO.html). KOBAS 2.0 (http://kobas.cbi.pku.edu.cn) was used for the enrichment analysis of DEGs in the KEGG pathways [44]. All the heatmaps were plotted with MeV 4.9.0 software (http://www.mybiosoftware.com/mev-4-6-2-multiple-experiment-viewer.html) based on the log_2_-transformed FRKM values. The expression values for a given gene were normalized for each row. All the transcriptome data was deposited in the NCBI SRA database under accessions SRR8477017-SRR8477022 and SRR8477029-SRR8477038.

### 2.3. Small RNA Data Analysis

Small RNA raw data was filtered with the SOAPnuke (https://github.com/BGI-flexlab/SOAPnuke) [45]. After removing the low-quality and contamination reads, only the reads longer than 18 nt or shorter than 30 nt were retained and aligned to unique sequences in the Rfam 14.1 database (http://www.sanger.ac.uk/software/Rfam) and the GenBank non-coding RNA database (http://www.ncbi.nlm.nih.gov/). The small RNAs were aligned to miRNA precursors in miRBase 21.0 (http://www.mirbase.org/) in order to assess the expression of known miRNAs in *B. napus* [46]. miRNAs were predicted by aligning with known miRNAs in miRBase. The relationship between target genes and miRNAs were predicted by an online program, psRNATarget (http://plantgrn.noble.org/psRNATarget/). Differentially expressed miRNAs were acquired, according to an online edgeR program (http://www.omicshare.com/tools/Home/Soft/diffanalysis), with default parameters. All the small RNA sequencing data were deposited in the NCBI SRA database under accessions SRR8284049-SRR8284056.

### 2.4. qRT-PCR Validation

Quantitative real-time PCR (qRT-PCR) was performed in a CFX96 Real-time System (Bio-RAD, USA) for validation of accuracy in the RNA-seq and miRNA profiling results. Total RNA was isolated using the same approach as described above. Briefly, 1 μg RNA from each sample was used to synthesize the single-stranded miRNA cDNA through reverse transcription using the miRcute miRNA first-strand cDNA synthesis kit (TIANGEN, Beijing, China) and miRNA-specific primers from this kit. Subsequently, the transcript levels of miRNAs involved in TPC formation were detected in samples from high- and low-TPC *B. napus* lines. Transcriptome results were validated by qRT-PCR using specific primers designed by the Primer Premier 5.0 (PREMIER Biosoft Int., Palo Alto, CA, USA) and the gene-specific primer database qPrimerDB (https://biodb.swu.edu.cn/qprimerdb/) [47]. All the qRT-PCR reactions were conducted using Novostar-SYBR Supermix (Novoprotein, Shanghai, China). The *ACTIN7* and the miRNA U6 in *B. napus* were used as internal controls to calculate the expression of target genes using the 2^−^*^ΔΔ^*Ct method. Each sample was subjected to three technical replicates. PCRs were performed with the following cycling parameters: 95 °C for 10 min, 40 cycles of denaturing at 95 °C for 30 s, annealing at 60 °C for 30 s, and extension at 72 °C for 30 s, followed by a disassociation stage (melting curve analysis). The threshold value was empirically determined based on the observed linear amplification phase of all primer sets. The sample cycle threshold (Ct) values were standardized for each template based on the actin gene control primer reaction [48].

## 3. Results

### 3.1. Identification of Differentially Expressed Genes by RNA-seq

TPC is a key trait of *B. napus*, which can influence other important abundant phenotypes, according to our investigation, such as economic yield (EY, grain weight per plant), plant height (PH), pod terminal height (PTH), stem height (SH), first effective branch height (FVBH), height of the lowest pod (POH), first effective branch number (FVBN), main inflorescence effective length (MIVL), and first effective branch number (FIBN) (Figure 1 and Table S2). The subjected plants used were taken from two sites on each plant, the stem apex, and a nearby tiny flower bud for transcriptome sequencing (Illumina HiSeq 2000, Illumina, San Diego, CA, USA), generating an average of ~43.47 million clean reads (39.96~50.53 million clean reads) from eight samples, (i.e., two sites from two low-TPC lines and two high-TPC lines) with two biological repeats. The mapping ratio of all the samples to the *B. napus* reference genome was approximate 83.9% [49].

**Figure 2:**
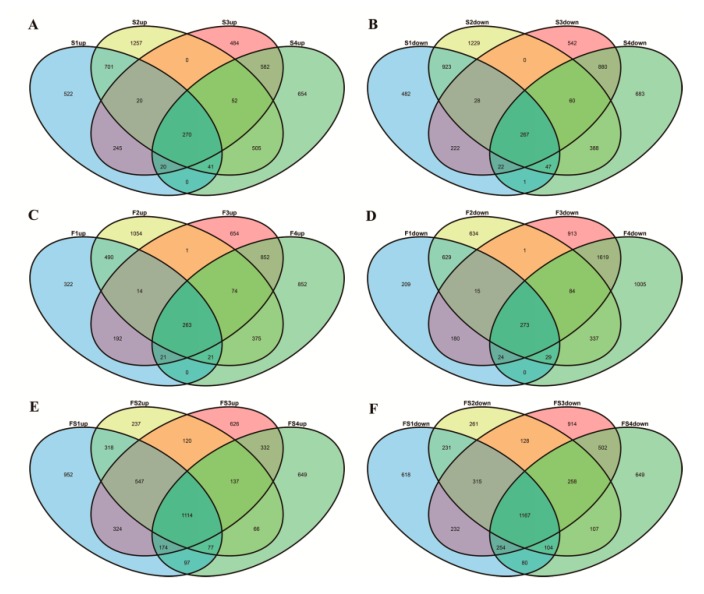
Differential expressed genes in high-TPC lines compared to low-TPC lines, according to RNA sequencing (RNA-seq). (**A**) Up-regulated differentially expressed genes (DEGs) between high- and low-TPC lines in the stem apex; (**B**) down-regulated DEGs between high- and low-TPC lines in the stem apex; (**C**) up-regulated DEGs between high- and low-TPC lines in the flower bud; (**D**) down-regulated DEGs between high- and low-TPC lines in the flower bud; (**E**) up-regulated consistently genes between stem apex and flower bud in the high- and low-TPC lines; (**F**) down-regulated consistently genes between stem apex and flower bud in the high- and low-TPC lines. S1, S2, S3, and S4 indicate the comparison results of YC4–YC11, YC4–YC15, YC33–YC11, and YC33–YC15 in the stem apex, respectively; F1, F2, F3, and F4 indicate the comparison results of YC4–YC11, YC4–YC15, YC33–YC11, and YC33–YC15 in the flower bud, respectively; FS1, FS2, FS3, and FS4 indicate the comparison results of YC4–YC11, YC4–YC15, YC33–YC11, and YC33–YC15, respectively, at two sites consistently.

In the stem apex samples, 3811 significant DEGs were detected between YC4 and YC11 with a fold change ≥2. We also identified 5788 DEGs between YC4 and YC15, 3694 DEGs between YC33 and YC11, and 4472 DEGs between YC33 and YC15. Of these, 537 genes were consistently differentially expressed between samples taken from the stem apex of high-TPC (YC4 and YC33) and low-TPC (YC11 and YC15) plants (Figure 2A,B, Table S3).

In the flower bud samples, we found 2682 DEGs between YC4 and YC11, 4294 DEGs between YC4 and YC15, 5180 DEGs between YC33 and YC11, and 5829 DEGs between YC33 and YC15. Among these, 536 genes were consistently differentially expressed between samples taken from the flower buds of high- and low-TPC plants (Figure 2C,D, Table S3). In total, 355 genes were differentially expressed conformably at both sites. Among these, 179 genes were up-regulated and 176 were down-regulated in high- as compared to low-TPC samples (Table S3). Furthermore, we found 6604, 5187, 6744, and 5767 DEGs between the stem apex and flower bud of YC4, YC11, YC15, and YC33, respectively. Of these, 2281 genes with same expression tendency showed a common differential expression trend at the two sampling sites; flower bud and stem apex (Figure 2E,F). Among these genes, 1114 were up-regulated and 1167 were down-regulated in the flower bud compared to the stem apex (Table S4).

### 3.2. Functional Classification by Gene Ontology (GO) and Metabolic Pathway Analysis

**Figure 3:**
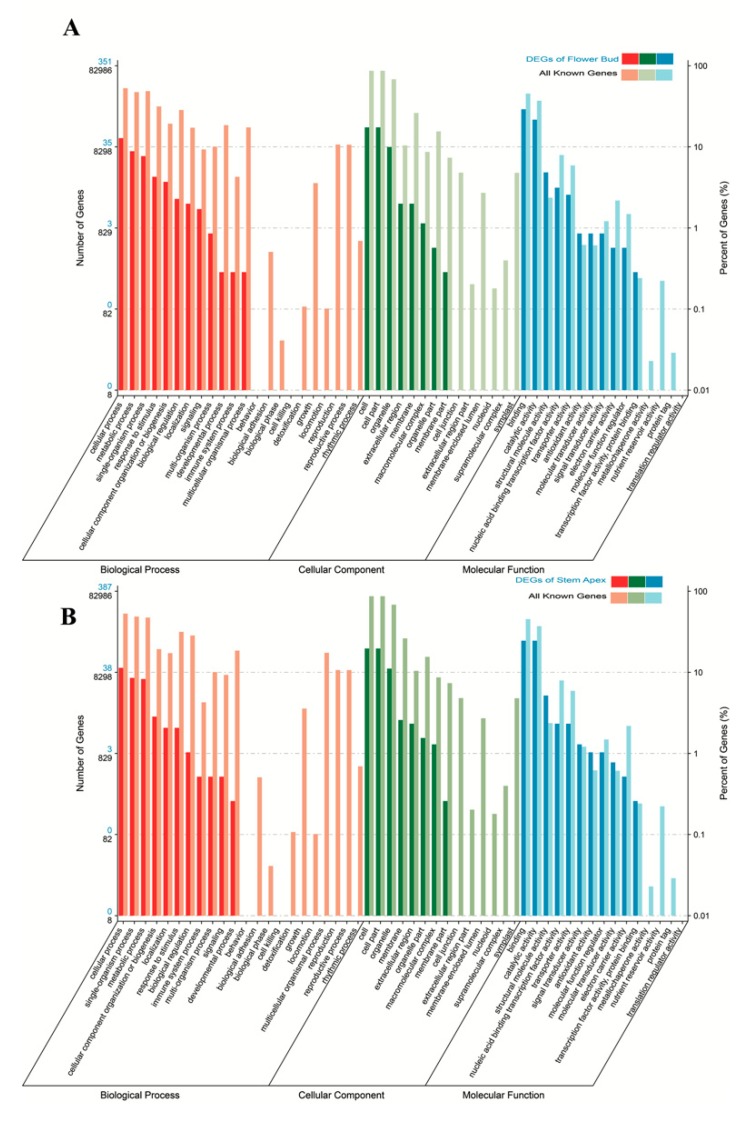
Gene ontology (GO) annotation analyses of DEGs identified at flower bud (**A**) and stem apex (**B**). Significantly, DEG distributions of the cellular component, molecular function, and biological process are almost consistent at the flower bud and stem apex.

Gene ontology (GO) annotation of the 536 and 537 DEGs identified at the flower bud and stem apex, respectively, between the two groups of samples showed that these DEGs were distributed into 51 different GO terms, which could be categorized into three main classifications: Cellular component (26% and 26%), molecular function (35% and 34%), and biological process (39% and 40%) (Figure 3A,B). We performed GO enrichment analysis to identify the over-enriched biological functions of these DEGs using a hypergeometric test.

**Figure 4:**
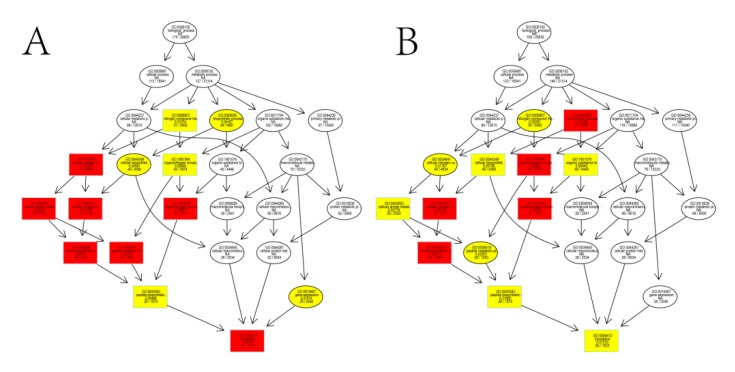
The noteworthy GO terms for both the flower bud (**A**) and stem apex (**B**). DEGs pertained to functions related to nitrogen compound biosynthesis and metabolism.

The *P*-values obtained were subjected to correction based on the FDR, and significant GO terms of differentially expressed genes were defined as those with a corrected *P* value of ≤0.05. A detailed analysis of the results indicated that almost all of the noteworthy GO terms for both the flower bud and stem apex were involved in the nitrogen compound biosynthesis and metabolism (Figure 4,B, Tables S5 and S6). 

**Figure 5:**
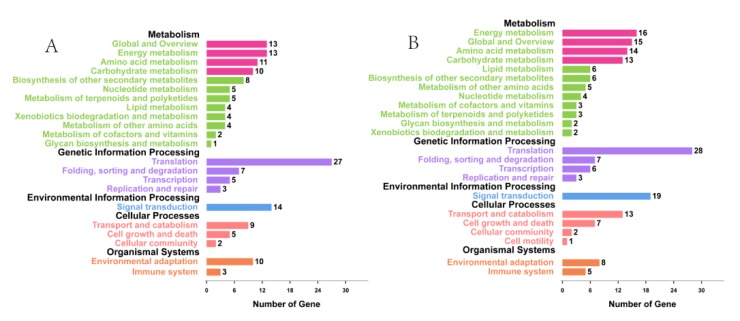
The Kyoto Encyclopedia of Genes and Genomes (KEGG) enrichment analyses of DEGs from the flower bud (**A**) and stem apex (**B**).

Subsequently, we mapped all the DEGs between high- and low-TPC plants from flower bud and stem apex to the KEGG database. The metabolic pathways of these DEGs were classified into 33 KEGG orthology (KO) terms (Figure 5A,B). KEGG enrichment analysis identified 18 and 20 significantly enriched pathways (Tables S7 and S8). Among these, energy metabolism (global and overview), amino acid metabolism, and carbohydrate metabolism pathways were the most abundant and were over-represented in both the flower bud and stem apex samples.

**Figure 6:**
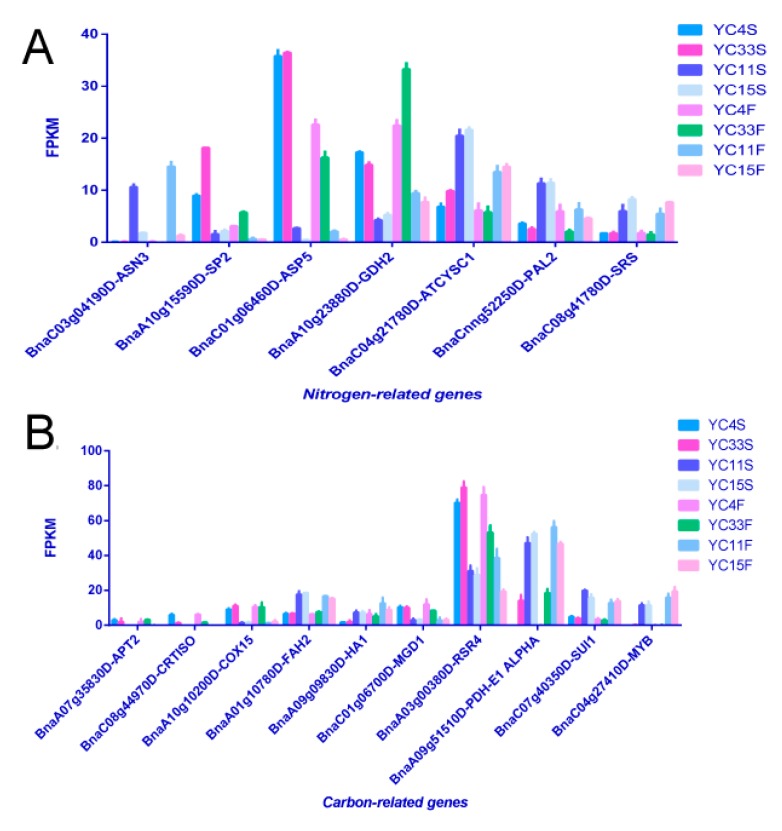
Many DEGs as key enzymes related to the nitrogen (**A**) and carbon (**B**) metabolisms. The expression value on the y-axis is derived from the fragments per kilobase of exon per million fragments mapped (FPKM) of RNA-Seq data.

A functional analysis of the DEGs was performed in the significantly enriched pathways in *B. napus* by mapping them to the *Arabidopsis* *thaliana* genome, and revealed that many DEGs are key enzymes involved in nitrogen and carbon metabolism. Four *B. napus* homologs of the *A. thaliana* genes *ASP5*, *ASP2,* and *ASN3*, which encode aspartate aminotransferases and an asparagine synthetase, were differentially expressed between high-TPC (YC4 and YC33) and low-TPC (YC11 and YC15) samples from both sampling sites (Figure 6A). Three homologs of *ATCYSC1*, *PAL2*, and *SRS*, which encode cysteine synthase c1, phenylalanine ammonia-lyase 2, and seryl-tRNA synthetase, respectively, in *A. thaliana*, were down-regulated in stem apex samples from high- as compared to low-TPC plants (Figure 6A). *GDH2*, a gene encoding an important nitrogen metabolism enzyme, glutamate dehydrogenase 2, was up-regulated in the stem apex and flower bud samples from both groups of plants, as was another homolog of the amino acid dehydrogenase protein family (Figure 6A).

Furthermore, some homologs of genes related to carbon metabolism or photomorphogenesis, such as *APT2*, *CRTISO*, and *COX15*, were up-regulated in high-TPC compared to low-TPC samples from both sites (Figure 6B). Two homologs of *FAH2* and *HA1* were down-regulated in the high-TPC samples from the stem apex, while *MGD1* and *RSR4* were up-regulated (Figure 6B). The homolog of a key gene encoding pyruvate dehydrogenase E1 α (PDH-E1 α) was down-regulated in the high-TPC samples from both sites (Figure 6B). Some homologs of important transcription factors related to morphogenesis, such as *SUI1* and *MYB*, were differentially expressed between the two classes of samples (i.e., high- and low-lines) from both sites (Figure 6B).

### 3.3. MiRNAs Differentially Expressed in High- and Low-TPC Samples

**Figure 7:**
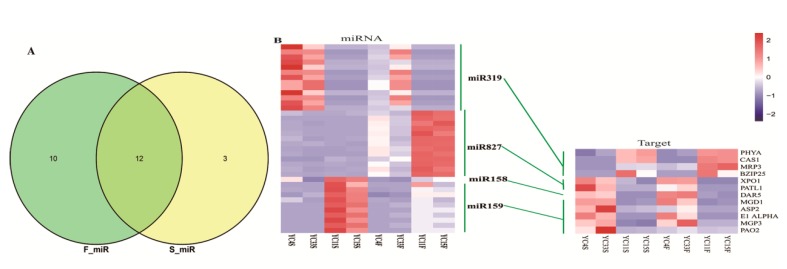
Identification of abundant known microRNAs (miRNAs) (**A**) and those regulating genes related to the nitrogen and carbon metabolisms (**B**).

Since plant phenotypic traits, such as height and branch angle, are influenced by the negative regulation of miRNAs, we also conducted small RNA sequencing to high- and low-TPC samples at both developmental sites. After removing adaptors and low-quality reads, we obtained ~13.33 and 15.93 million (YC4), ~13.95 and 13.63 million (YC11), ~15.25 and 13.39 million (YC15), and ~15.25 and 14.99 million (YC33) clean reads, respectively, from the flower bud and stem apex, respectively. Comparing miRNA expression on between high- and low-TPC samples identified 22 and 15 differentially expressed known miRNAs at the flower bud and stem apex, respectively (Figure 7A, Table S9). Furthermore, we identified a total of 929 novel miRNAs (Table S10), among which 7 were differentially expressed between the two sets of samples (Table S10).

Of 31 sequences that were differentially up-regulated in high-TPC compared to low-TPC samples from the stem apex, we identified 22 as members of the miR319 family. Five up-regulated sequences were members of the miR6029 family. The other up-regulated miRNAs at the stem apex were identified as miR159, miR6030, miR9409, and miR9563. Of 26 sequences that were down-regulated at the stem apex, four were identified as miR158. Six miRNAs were found to be homologs of miR159, a conserved miRNA. Five miRNAs were identified as members of the miR845 family. The other up-regulated miRNAs were identified as homologs of miR10, miR122, miR1511, miR169, miR319, miR5726, miR6030, miR6483, and miR9410.

At the flower bud, 45 sequences showed down-regulation. Of these sequences, 18 were identified as members of the miR827 family. Five down-regulated miRNAs were identified as miR159 and four as miR122. The other down-regulated miRNAs were identified as miR10, miR143, miR148, miR1511, miR158, miR167, miR192, miR319, miR395, miR6030, miR845, and miR9410. In addition, we found 20 miRNAs that were significantly up-regulated in the flower bud. Four miRNAs were identified as miR166f. The other miRNAs were identified as miR1140, miR159, miR165, miR319, miR5153, miR5726, miR9409, and miR9563.

### 3.4. Validation of Differential Gene and miRNA Expression by qRT-PCR

**Figure 8:**
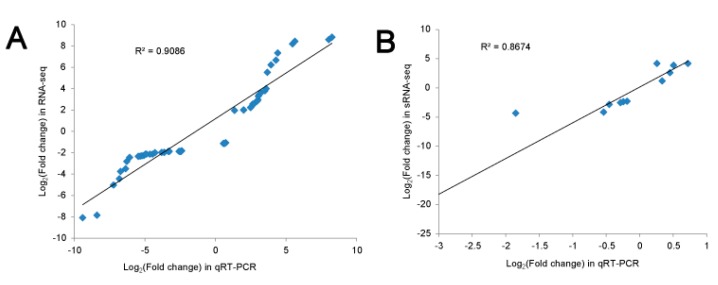
Comparison of gene expression values obtained from RNA-seq, sRNA-seq and quantitative real-time PCR (qRT-PCR) results. (**A**) Log_2_ (fold change) were calculated for 17 DEGs, and a high correlation (*R*^2^ > 0.93, *P* < 0.01) was observed between RNA-seq and qRT-PCR results. (**B**) Similarly, Log_2_ (fold change) for 12 differentially expressed miRNAs were calculated, and the correlation (*R*^2^ > 0.86, *P* < 0.01) was showed between sRNA-seq and RT-qPCR results.

To test the reliability of the RNA-seq results, we randomly selected 17 DEGs (including 5 from the flower bud and 12 from the stem apex) for qRT-PCR detection. As expected, the qRT-PCR results showed high consistency with the RNA-seq data (Figure 8A, Tables S11 and S12). The Pearson correlation coefficient between the log_2_ (fold change) of the RNA-seq and qRT-PCR results was 0.9086 (*p* < 0.01, two-tailed). We also conducted qRT-PCR to validate the accuracy of the small RNA sequencing (sRNA-seq) results for 12 randomly selected miRNAs (6 from the flower bud and 6 from the stem apex). The Pearson correlation coefficient between the sRNA-seq and qRT-PCR results was 0.8674 (*p* < 0.01, two-tailed) (Figure 8B, Tables S13 and S14). Therefore, the sequencing results of both RNA-seq and sRNA-seq are highly correlated with qRT-PCR, which indicates that our data is accurate and reliable and can be used for further analysis.

### 3.5. MiRNA and Gene Network Analysis in High- and Low-TPC Samples

The expression levels of the majority of miRNAs were negatively associated with those of the corresponding target genes. When the expression of differentially expressed miRNA was higher in high-TPC samples than in low-TPC samples, the expression of some potential target genes was lower. For instance, miR319 was up-regulated in the high-TPC lines compared to low-TPC plants, and the expression of most of its target genes, such as *PHYA*, *CAS1*, *MRP3*, and *BZIP25*, was decreased. We also performed joint analyses of other representative miRNAs. We found that the expression of miR827 was down-regulated in flower bud samples from high-TPC plants than low-TPC plants, and the target genes of miR827, EXPOTIN 1 (*XPO1*) and PATELLIN 1 (*PATL1*), correspondingly showed increased expression. Likewise, DA1-RELATED PROTEIN 5 (*DAR5*), MONOGALACTOSYL DIACYLGLYCEROL SYNTHASE 1 (*MGD1*), ASPARTATE AMINOTRANSFERASE 2 (*ASP2*), PYRUVATE DEHYDROGENASE COMPLEX E1 α SUBUNIT (*E1 α*), MALE GAMETOPHYTE DEFECTIVE 3 (*MGP3*), and POLYAMINE OXIDASE 2 (*PAO2*) were predicted to be gene targets of miRNA158 and miR159. These two miRNAs displayed decreased expression in stem samples from high-TPC compared to low-TPC plants, while the target genes were up-regulated (Figure 7B). These results suggest that our miRNA profiling and RNA-seq data was reliable. Moreover, the miRNA-gene pairs with inverse transcriptional associations may be useful as potential candidates for further genetic manipulation of plant TPC-related traits.

## 4. Discussion

The TPC is mainly determined by plant height (PH), pod terminal height (PTH), stem height (SH), first effective branch height (FVBH), pod origin height (POH), first effective branch number (FVBN), main inflorescence effective length (MIVL), and first effective branch number (FIBN), as described above. These traits are related to plant architecture and aboveground shape and are controlled by endogenous and environmental factors [50,51,52]. Therefore, TPC is an important trait determining ideal plant architecture and yield in *B. napus* as well as in other crops. Recently obtained results showed that using a planting density of 30 plants m^−2^ can improve yield and nitrogen use efficiency and enhance resistance to lodging by improving the crop canopy [53]. However, the underlying mechanisms of pod canopy regulation are still largely not understood. So far, few direct studies have been done of the factors influencing the thickness of the pod canopy. With the development of high-throughput sequencing technology, it is now relatively easy to identify genes that are differentially expressed between plants with different canopy shapes. In this study, we performed RNA and miRNA profiling in order to ascertain the molecular mechanisms underlying the regulation of pod canopy thickness in *B. napus*, and we identified numerous DEGs between two groups of cultivars with different thicknesses of pod canopies. These included key genes involved in the biosynthesis and signaling transduction of nitrogen. This provides substantive information related to possible strategies for establishing ideal plant architecture in *B. napus*.

Both the KEGG and GO enrichment analyses results showed that noteworthy GO terms mainly associated with the nitrogen compound biosynthesis and metabolism influencing TPC variation in *B. napus*. It has been proven that nitrogen and carbon metabolisms are coordinated with each other in many important biological processes in plant development. On the one hand, the nitrogen metabolism and uptake requires cotransporters, reductants, energy carriers, and carbon skeletons that are derived from the carbon metabolism [54,55,56,57]. On the other hand, crucial carbon metabolic pathways, including mitochondrial respiration, photorespiration, and photosynthesis, are dependent on nitrogen availability [56]. Recent studies have shown that nitrogen rate (the amount of nitrogen available to the plants) significantly influences yield- and TPC-related traits, such as plant height, pod area index, and shoot number in *B. napus* [53], implying that the nitrogen metabolism may influence the *B. napus* yield through carbon metabolism regulation.

TPC is a complicated comprehensive trait that encompasses multiple plant phenotypic traits, including plant height, stem height, pod terminal height, pod original height, main inflorescence length, shoot, and so on. Previous studies have indicated that nitrogen and phosphorus fertilization negatively affects SL production and exudation in sorghum and further regulates shoot and root architecture [58]. These two nutrients can also significantly affect plant height and stem height [53,59]. Thickness of pod canopy is mainly influenced by environmental and internal nutritional factors [60,61]. Final organ size and dimensions are governed by cell division, which is dependent on hormones, particularly auxin, abscisic acid (ABA), and CKs, but also on the status of carbon and nitrogen [61,62,63,64,65]. Therefore, TPC as a plant architectural trait is the result of a complicated web of interactions between phytohormone and nutritional/metabolic signaling pathways [66]. Studies recently found that the amine/nitrate ratio interacts with GA signaling and respiratory pathways and further regulates the partitioning of biomass between shoots and roots [67]. In fact, nitrogen and related nutrients contribute significantly to the formation of valid pods and to further changing of pod height [53]. Normally, high abundance of nutrients, such as nitrogen and carbon, play an important role in the production of effective pods. In our study, many genes encoding nitrogen and carbon biosynthesis and signaling transduction were found to be significantly differentially expressed in high-TPC compared to low-TPC plants. Therefore, we speculate that the TPC is modulated by changes in nitrogen and carbon metabolism pathways and further influences TPC-related traits. Further genetic studies need to be conducted to establish the detailed function of these genes and their protein products in the key steps of the carbon biosynthesis or nitrogen signaling pathways.

miRNAs play a critical role in the formation of plant architecture. Recently, miR319 was proposed to be involved in regulating plant height, possibly by regulating the elongation of internodes, which leads to decreased plant height in rice [34]. Auxin and BR play an important role in the regulating branch angle of *B. napus* [38]. Expression patterns of most target genes involved in auxin- and BR-related pathways have been found to be fine-tuned by related miRNAs, such as miR156, miR172, and miR319 [38]. Overexpression of rice Os-miR160 decreases the effective tiller number while increasing the tiller angle [68].

In this study, a comparison of miRNA expression in high- and low-TPC samples identified 22 and 15 miRNAs differentially expressed in the flower bud and stem apex, respectively. Many of the miRNAs identified are directly related to the regulation of plant architecture or influence plant phenotype through regulation of nitrogen and carbon metabolisms. MiR319 and miR6029 were differentially up-regulated in high-TPC compared to low-TPC samples from the stem apex. The former was recently demonstrated to play an important role in suppressing rice plant height [34]. MiR6029 is involved in *B. napus* amino acid metabolism [69], oil production [70], and fatty acid biosynthesis [71]. MiR158, miR159, and miR845 were down-regulated at the stem apex. MiR158 has been proven to regulate pollen development in non-heading Chinese cabbage (*Brassica campestris* ssp. *chinensis*) [72]. MiR159 and miR845 are involved in the control of stem length in rice [73] and crop resistance [74,75], respectively. MiR827 showed down-regulation at the flower bud, which may target genes encoding ubiquitin E3 ligase with RING and SPX, and further influence nitrogen and phosphorus metabolisms [76]. MiR166f was significantly up-regulated in the flower bud, which has been found to regulate the branch angle in *B. napus* [38]. Overall, many of the putative miRNAs we identified as differentially regulated between high- and low-TPC samples in this study have previously been found to be involved in the nitrogen metabolism, such as miR159, miR166, miR167, miR319, miR395, and miR827 [76].

Furthermore, as has been suggested by other studies [77,78], we found that one miRNA may affect multiple target genes, and, conversely, one gene may be regulated by multiple miRNAs. miR319a was more abundant in low-TPC samples than in high-TPC samples at the bolting with tiny flower bud stage, while the expression of its predicted target genes *PHYA* and *BZIP25* was down-regulated in low- as compared to high-TPC samples from both sites; both genes play an important role in influencing photomorphogenesis, efficiency of phosphorus utilization, and seed development [79,80,81,82]. We also found that numerous other genes predicted to be related to nitrogen and carbon pathways, such as *PAL2* [83], *KIN10* [84], and *CAS1* [85], were differentially expressed between the two groups based on the differential expression of miR319a. Members of the miR827 family were up-regulated in flower buds of high-TPC as compared to low-TPC plants, while their target genes *AMK2*, *XPO1A*, *PATL1*, and *KNAT5* were down-regulated. The products of these genes have functions related to carbon metabolism, floral organ development, and shoot apical meristem development [86,87,88,89]. Indeed, many genes were detected with products involved in carbon- and nitrogen-related pathways that were expressed differentially between the two groups of samples as a result of changes in miRNA expression: For instance, *PK1* for miR165, *ASN3* for miR5153, *PGIP1* for miR5726, *ASP2* for miR122 and miR159, ARF8 and *GPX8* for miR395d, *IAA18* and *CRTISO* for miR6030, and *GRF8* and *AAO1* for miR159. Moreover, numerous miRNAs related to regulating plant architecture, such as miR156, miR159, miR319, miR169, and miR827 [34,38,73,76,90], were differentially expressed between high- and low-TPC samples. Therefore, the regulation mechanism of TPC, as an important agronomic trait related to multiple miRNAs, warrants further analysis. Subsequently, some important miRNAs derived from this study will be selected to construct overexpression vectors and observe phenotypic changes related to TPC traits through Arabidopsis and rapeseed transformation. Recently, studies in *B. napus* have shown that methylation and small RNA are involved in the regulation of traits simultaneously [91]. Hence, further methylation analysis of our remaining samples, combined with the results obtained in this study, will be more helpful for a deeper understanding of the genetic mechanism of TPC traits.

## Supplementary Materials

The following are available online at https://www.mdpi.com/2073-4425/10/8/591/s1, Table S1: The average TPC values of four varieties in continuous three years. Table S2: The correlations analysis of plant architecture in *B. napus*. Table S3: Differentially expressed genes in high-TPC lines compared to low-TPC lines. Table S4: Differentially expressed genes between two sites in all sequenced samples, Table S5: Enriched GO terms of DEGs in the flower bud. Table S6: Enriched GO terms of common DEGs in the stem apex. Table S7: Enriched KEGG pathway of DEGs in the flower bud. Table S8: Enriched KEGG pathway of DEGs in the stem apex. Table S9: Differentially expressed miRNAs in the stem apex and flower bud. Table S10: Novel miRNAs detected in the high- and low-TPC lines. Table S11: Result comparison of RNA-Seq and qRT-PCR for randomly selected genes. Table S12: qRT-PCR primers used for validation of expression pattern of selected genes. Table S13: Result comparison of sRNA-Seq and qRT-PCR for randomly selected miRNAs. Table S14: qRT-PCR primers used for validation of expression pattern of selected miRNAs.
